# Prognostic prediction of clear cell renal cell carcinoma based on lipid metabolism-related lncRNA risk coefficient model

**DOI:** 10.3389/fgene.2022.1040421

**Published:** 2023-01-04

**Authors:** GenYi Qu, Lu Liu, Lai Yi, Cheng Tang, Guang Yang, Dan Chen, Yong Xu

**Affiliations:** ^**1**^ Department of Urology, ZhuZhou central Hospital, ZhuZhou, China; ^2^ Department of Ultrasound, ZhuZhou central Hospital, ZhuZhou, China; ^3^ Department of Hematology, ZhuZhou central Hospital, ZhuZhou, China

**Keywords:** lipid metabolism, clear cell renal cell carcinoma, lncRNA, risk model, prognosis

## Abstract

**Objective:** In order to predict the prognosis in patients with clear cell renal cell carcinoma (ccRCC) so as to understand cancer lipid metabolism and sensitivity to immune-targeting drugs, model algorithms were used to establish a risk coefficient model of long non-coding RNAs (lncRNAs) associated with lipid metabolism.

**Methods:** The transcriptome data were retrieved from TCGA, and lncRNAs associated with lipid metabolism were obtained through Pearson correlation and differential expression analyses. Differentially expressed lipid metabolism-related lncRNAs and lipid metabolism-related lncRNA pairs were obtained using the R language software. The minimum absolute shrinkage method and the selector operation regression method were used to construct the model and draw the receiver operator characteristic curve. High-risk patients were differentiated from low-risk patients through the cut-off value, and the correlation analyses of the high-risk subgroup and low-risk subgroup were performed.

**Results:** This research discovered that 25 pairs of lncRNAs were associated with the lipid metabolism of ccRCC, and 12 of these pairs were utilized to build the model. In combination with clinical data, the areas under the 1-, 3- and 5-year survival curves of ccRCC patients were 0.809, 0.764 and 0.792, separately. The cut-off value was used to perform subgroup analysis. The results showed that high-risk patients had poor prognosis. The results of Cox multivariate regressive analyses revealed that age and risk score were independent prediction factors of ccRCC prognosis. In addition, immune cell infiltration, the levels of gene expression at immune checkpoints, and high-risk patients more susceptible to sunitinib-targeted treatment were assessed by the risk model.

**Conclusion:** Our team identified new prognostic markers of ccRCC and established risk models that could assess the prognosis of ccRCC patients and help determine which type of patients were more susceptible to sunitinib. These discoveries are vital for the optimization of risk stratification and personalized management.

## Introduction

Renal cell carcinoma (RCC) is one of the most commonly seen malignancies of the urinary system, taking up 2%–3% of the entire malignancies (Mao et al., 2021; Mondlane et al., 2021; Turco et al., 2021). Recently, the prevalence of RCC has been elevating. In China, the morbidity of RCC increased from 3.96 per 100,000 in 2005 to 9.47 per 100,000 in 2012, and the incidence of RCC in all stages was elevated as well (Zhang et al., 2021a; Kubiliute and Jarmalaite, 2021; Xu et al., 2021). ccRCC is the most commonly seen histologic sub-type of RCC, taking up 75%–80% of all kidney cancer cases (Xiao and Meierhofer, 2019; Christensen et al., 2021). Surgery is the main therapeutic method for ccRCC, and the surgical resection of localized ccRCC usually extends the progression-free survival and OS of sufferers (Curtis et al., 2016). However, about 20%–30% of sufferers have metastatic renal cell carcinoma at the time of diagnosis. In addition, 30% of newly diagnosed locally advanced renal cancer patients will suffer from metastases (Bindayi et al., 2018). Even with the remarkable progress in diagnosis and treatment strategies based on tyrosine kinase inhibitors and target rapamycin inhibitors, clinical outcomes in patients with renal cancer are still not satisfactory due to drug resistance and side effects (Choueiri et al., 2015; Matrana et al., 2016). Hence, it is vital to reveal the potential molecule-level causal link of malignant ccRCC and develop new drug targets and therapeutic strategies.

At present, more and more evidences show that kidney cancer is a metabolic disease. This kind of metabolic abnormality not only supports the synthesis of protein, lipid and nucleic acid macromolecules, but also facilitates the proliferative and invasive abilities of oncocytes (Wettersten et al., 2017; Ma et al., 2021). The variations of lipid metabolism, especially the synthetic membrane compositions of fatty acid and cholesterol metabolism, are vital for tumor occurrence, development, fatty acid, cholesterol in the cell lipid metabolism is a flexible, feedback loops and crosstalk access network, through the mutual adjustment metabolism of cancer cells, to meet the increasing demand (Arendowski et al., 2021; Zhang et al., 2021b).

With the development of biotechnology, the results of the Human Genome Project show that less than 2% of the nucleic acid sequence is used to encode proteins, and the rest part doesn’t express proteins, which are called non-coding RNAs(ncRNA) (He et al., 2021; Yip et al., 2021; Zhou et al., 2021). ncRNA was originally thought to be transcriptional noise, but with the advancement of researches, it is been discovered that ncRNA is pivotal for normal development and disease progression (Bao et al., 2021; Chen et al., 2021; Smolarz et al., 2021). ncRNAs exceeding 200 nt are called lncRNAs. lncRNA can mediate gene regulation by interacting with DNA, RNA or protein, and its mechanism involves transcriptional regulation and post-transcriptional regulation that affect the activity of genes encoding proteins. It can directly combine with some proteins to form nucleic acid protein complexes, which plays a regulatory role by affecting the localization of proteins in cells, and it involves multiple pathways closely related to cancer development and progression, such as p53, NF-κB, PI3K/AKT and Notch (Huang et al., 2021; Baumann, 2022; Reggiardo et al., 2022). Nevertheless, there are insufficient researches on the regulation of lipid metabolism by lncRNA in renal cancer. Therefore, the present paper intends to establish a risk coefficient model of lncRNA associated with lipid metabolism through model algorithm, lncRNA pairing and iteration, so as to forecast the prognoses of ccRCC sufferers and understand the lipid metabolism and the sensitivity of targeted drugs.

## Materials and methods

### Data collection

We used the GDC DataTransfer Kit to acquire publicly available transcriptomic data of ccRCC and paracancer normal tissue from TCGA (https://cancergenome.nih.gov/) (Tomczak et al., 2015), which involved 539 ccRCC specimens and 72 paracancer normal tissue specimens. Next, our team used Ensembl (http://asia.ensembl.org) (Yates et al., 2020) to download the Gene Transfer Format (GTF) files to annotate and differentiate the mRNAs and lncRNAs in transcriptome data. The list of lipid metabolism-related genes was download from the publicly available MSigDB database (Subramanian et al., 2005).

### Analysis of differentially expressed lncRNA correlated to lipid metabolism

Based on the coexpression correlative coefficient >0.7 and *p* < 0.001, the coexpression strategy and Pearson correlative analysis were utilized to obtain the lipid metabolism-associated lncRNAs. Differentially expressed lncRNAs correlated to lipid metabolism were selected by using the limma package of the R language software, and we set the parameters as |logFold Change| > 1.0 and FDR <0.05 (Ritchie et al., 2015). In addition, the visualization of the obtained lncRNAs was realized by using a HeatMap package.

### Construction of lncRNA pairs associated with lipid metabolism

Multiple rounds of pairing were utilized to identify differentially expressed lncRNAs correlated with lipid metabolism, and we took the parameter value 0 or 1 as the definition value and α as the parametric value. When the expressing level of lncRNA 1 was higher in contrast to lncRNA 2 in the lipid metabolism-associated lncRNA pairs, the α value of the lncRNA pair was 1. Otherwise, the value of α was 0. When the ratio of lncRNA associated with lipid metabolism to α value (0 or 1) was less than 80% in all samples, the lncRNA pairs associated with lipid metabolism were effectively matched. Otherwise, rematch was required.

### Clinical data acquisition and model establishment

Firstly, our team acquired clinical data of ccRCC from TCGA, and afterwards we paired lncRNA associated with lipid metabolism in the previous step *via* the limma package from the R program. Afterwards, the intersection was taken, while duplicate clinical information without follow up time was removed. Univariate regressive analyses were completed for the preliminarily obtained lipid metabolism-related lncRNA pairs to identify the lipid metabolism-related lncRNA pairs associated with survival status. *p* < 0.01 was the threshold of significance.

In order to avoid overfitting, we used the glmnet package to complete the second crossvalidation of lipid metabolism-related lncRNA pairs obtained from LASSO regression analysis (Engebretsen and Bohlin, 2019), and a 1000-repeat random cycle was finished, and the lipid metabolism-related lncRNA pairs with a matching frequency of over 100 times with *p* < 0.05. The optimal pairing combinations were selected to obtain the lipid metabolism-related lncRNA pairs involved in the construction of the Cox risk coefficient model. Cox univariable and multivariable models were constructed to determine the risk coefficients of each lipid metabolism-related lncRNA pair associated with outcomes and determine the risk score of every cancer specimen. The overall risk score for every specimen equalled the expression level of each lipid metabolism-associated lncRNA pair in the specimen multiplied by the sum of risk factors. The equation is as follows:

$$Risk\;Score=\overset{n}{\underset{i=1}{\sum }}Risk\;coefficient\times lipid\;metabolism-related\;lncRNA\;Expression$$

We visualized the Cox analysis outcomes with the survminer and survival packages in the R program.

### ROC curve was established by risk coefficient model

We used the survival ROC package of the R program to construct the 1-, 3-, and 5-year ROC curves, and AUC values were computed to identify the model-forecasted values. Our team discovered that the AUC value at 1 year was the largest. Finding the threshold at which the sum of specificity and sensitivity maximizes as per the AIC best fit makes it probable to differentiate risk_high_ and risk_low_ sufferers.

### Risk coefficient model was used to analyze clinical correlation

We used the survival and survminer packages of the R program to contrast survival diversities between risk_high_ and risk_low_ sufferers, and *p* < 0.001 had significance on statistics. The Kaplan-Meier (K-M) method was utilized to realize data visualization. Chi-square test was employed to study the correlation between risk score and the acquired clinical indexes (survival, age, gender, tumor grade, tumor stage, T, N, and M stage). Wilcoxon rank-sum test was utilized to reveal the correlation between risk score and diverse subgroups of clinical indicators. We used the limma and ggpubr packages of the R program to realize data visualization. For the sake of identifying if risk score can be utilized as an independent prediction factor for ccRCC sufferers, our team completed Cox univariable and multivariable regressive analyses of risk score and clinical indexes, and evaluated relevant hazard ratios. With *p* < 0.05 as the discrimination standard, the survival package of the R program was utilized realize data visualization. To contrast the accurateness of risk score and clinical indexes in forecasting OS and results, ROC curves acquired in the 1-year follow up were contrasted with ROC curves of clinical indexes in the identical curve.

### Association analyses of immune cells

To reveal the association better risky factor score and immune cell infiltration, the immune cell infiltration data of ccRCC sufferers in TCGA were computed as per CIBERSORT (http://cibersort.stanford.edu/), TIMER 2.0 (http://timer.cistrome.org/), QUANTISEQ (http://icbi.at/quantiseq), Micro-environment Cell Populations-counter, EPIC (http://epic.gfellerlab.org), and XCELL (http://xCell.ucsf.edu/). Our team utilized limma, scales, ggplot2, and ggtext packages of the R program to study the association between immune cell infiltration data and risk coefficient score, and visualized the bubble map with *p* < 0.05 as the discriminant standard.

### Association analyses of genes

Our team discovered that the expression levels of CD274, CTLA4, LAG3, LGALS9, PDCD1, PDCD1LG2, HAVCR2 and TIGIT were high in ccRCC. To identify if those genes differ between risk_high_ and risk_low_ sufferers, we analyzed and visualized the data using violin diagrams *via* the limma and GGpubr packages of the R program.

### Association analyses of target drugs

In order to confirmed if there is diversity in reaction to targeted drugs between risk_high_ and risk_low_ sufferers, the drug’s semi-inhibition rate (IC50) was utilized as an indicator of drug sensitivity. In addition, we analyzed and visualized the through the limma, ggpubr, ggplot, and pRRophetic packages.

## Results

**FIGURE 1:**
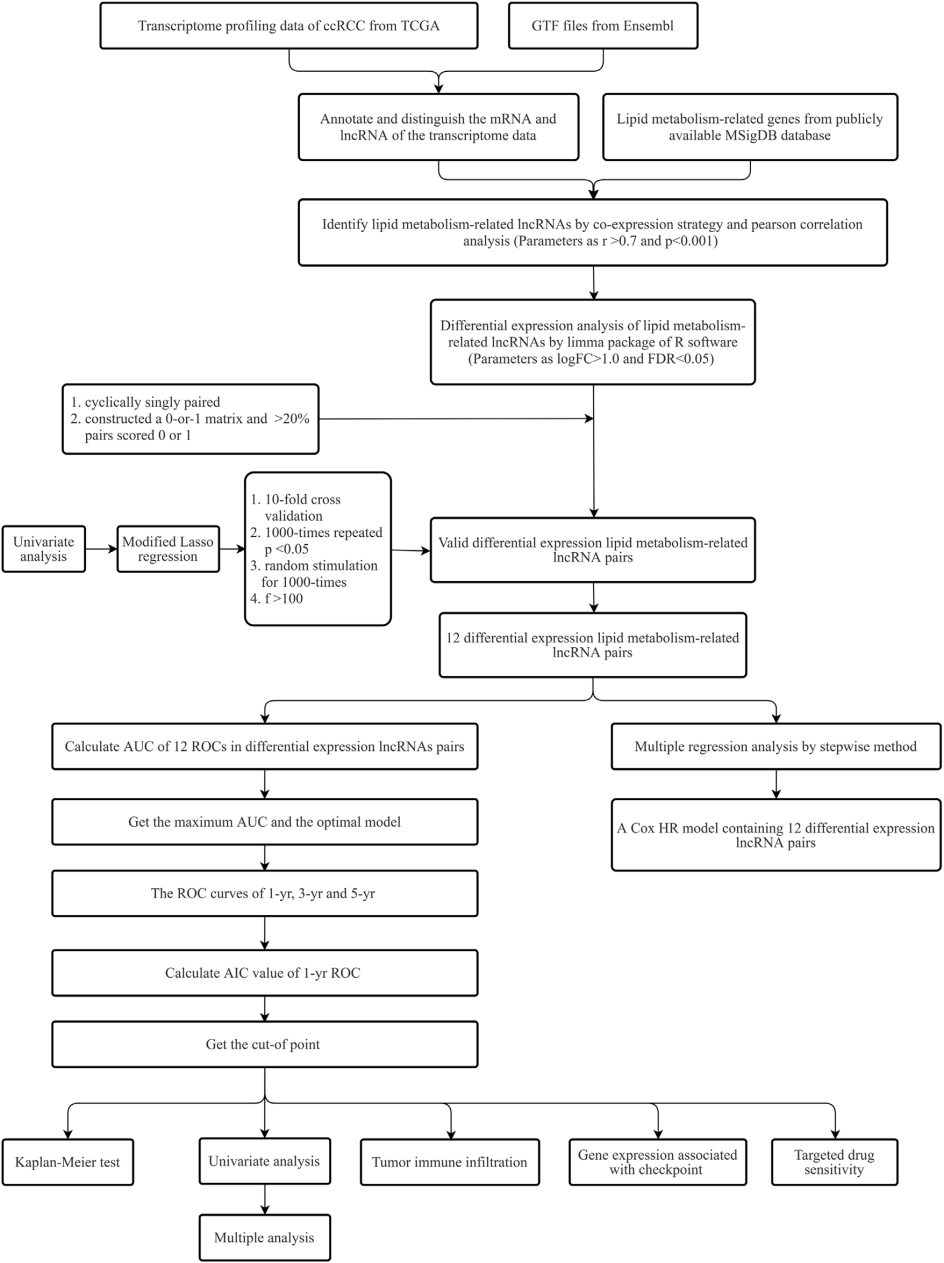
The process flow of the present research.

Figure 1 provides the process flow of the present research.

### Differential expression analysis of lncRNAs associated with lipid metabolism in ccRCC

**FIGURE 2:**
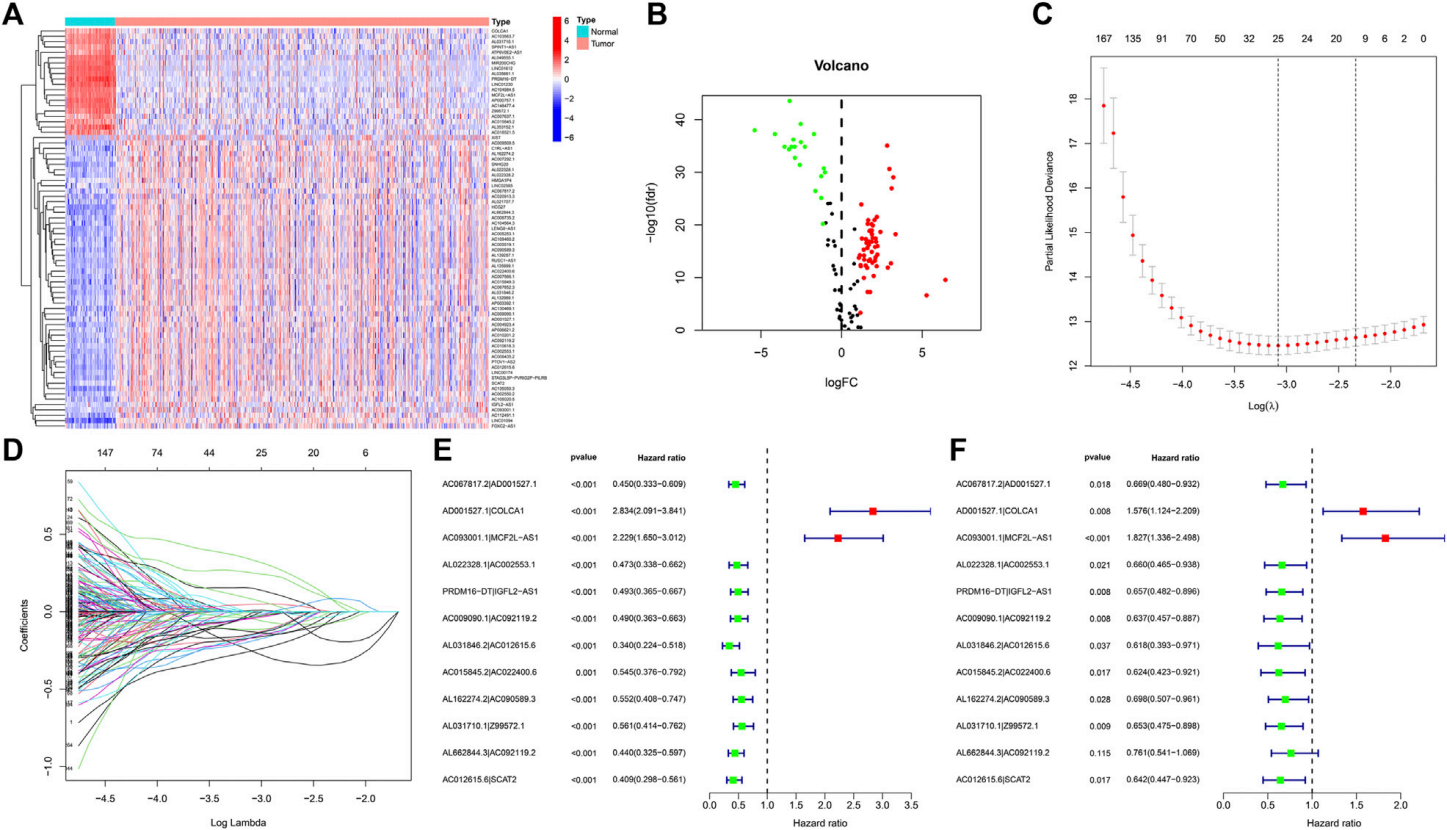
DE analysis of lncRNAs associated with lipid metabolism in ccRCC. **(A)** Heatmap of differentially expressed lipid metabolism lncRNAs. The red color denotes upregulation, and blue color denotes downregulation. **(B)** Volcanic map of differentially expressed lipid metabolism lncRNAs. Red dots: upward regulation with remarkable differential expression; green dots: downward regulation with remarkable differential expression; black dots: no remarkable diversity. **(C)** Elucidation of LASSO coefficient profling of those prognosis-related lipid metabolism lncRNAs. **(D)** Verification of tuning parameter selection for LASSO regressive model. **(E)** Univariable cox regressive analysis of the aberrantly regulated lipid metabolism-associated lncRNAs which might remarkably influence the OS of ccRCC sufferers. Red: risky factor; Green: protection factor. **(F)** Multivariable Cox regressive analysis of promising prognosis lipid metabolism-associated lncRNAs.

Transcriptome and lipid metabolism information of ccRCC was acquired from the TCGA data base. We annotated and differentiated transcriptome data through the Ensembl data base. A total of 111 lncRNAs related to lipid metabolism were determined *via* Pearson correlative analyses, with coexpression correlative coefficient >0.7 and *p* < 0.001 as the identification standard (Supplementary Table S1). We adopted differential expression analyses, with |log a FC| > 1.0 and FDR <0.05 being the identification standards. Eventually, 75 differentially expressed lipid metabolism lncRNAs were determined and our team used R software to generate the gene heat map (Figure 2A). In ccRCC, 20 lncRNAs were regulated downward and 55 lncRNAs were regulated upward (Figure 2B and Table 1).

**TABLE 1:** Lipid metabolism-related lncRNAs acquired posterior to differential expression analyses.

lncRNA	conMean	treatMean	logFC	*p*-value	FDR
AC067817.2	0.250253	0.993011	1.988419	3.68E-11	4.97E-11
AC104984.5	3.682574	0.462925	−2.99186	3.54E-38	6.55E-37
PTOV1-AS2	0.642868	2.771483	2.108063	1.53E-18	3.78E-18
AC020913.3	0.074073	0.617973	3.060524	1.27E-13	1.98E-13
AD001527.1	0.127594	0.576004	2.174521	4.91E-13	7.11E-13
AC009509.5	0.625893	1.369603	1.12977	3.24E-15	5.81E-15
AL139287.1	2.075307	5.486949	1.40268	2.36E-14	3.95E-14
AC093001.1	0.011144	0.96597	6.437666	2.11E-10	2.79E-10
AC103563.7	5.173696	0.597978	−3.11303	1.51E-36	1.47E-35
HMGA1P4	0.423841	0.952361	1.167984	0.000449	0.000503
AL022328.1	0.488095	1.278296	1.388989	3.64E-15	6.40E-15
PRDM16-DT	13.2686	0.316472	−5.3898	2.97E-40	1.10E-38
AC009090.1	0.253848	0.813873	1.680838	3.89E-14	6.27E-14
AL049555.1	3.540425	0.370258	−3.25732	5.29E-36	4.51E-35
AL021707.7	0.12328	0.576657	2.225772	5.35E-17	1.06E-16
AC007637.1	2.486031	1.112996	−1.1594	1.88E-21	6.31E-21
C1RL-AS1	1.378692	4.238105	1.620119	1.84E-21	6.31E-21
LINC01230	2.447813	0.748823	−1.7088	2.60E-39	5.77E-38
FOXC2-AS1	0.099379	0.724179	2.865338	9.22E-13	1.31E-12
AL031846.2	0.165473	0.609877	1.881926	2.26E-19	5.97E-19
AC015845.2	1.708304	0.836163	−1.03071	1.83E-31	1.13E-30
RUSC1-AS1	0.482382	1.460942	1.59865	1.31E-16	2.50E-16
AL162274.2	0.75212	1.582993	1.073619	1.05E-14	1.79E-14
AC112491.1	0.3593	1.37393	1.935047	3.83E-21	1.22E-20
AL031710.1	10.23424	1.697658	−2.59179	5.68E-33	4.20E-32
AC005253.1	0.241345	0.827065	1.776902	4.04E-20	1.18E-19
AC008735.2	0.609316	3.23721	2.409489	7.61E-20	2.11E-19
MCF2L-AS1	2.411878	0.498238	−2.27525	1.59E-36	1.47E-35
XIST	0.997847	3.059421	1.616367	4.48E-08	5.52E-08
AL662844.3	0.115057	0.989206	3.103922	2.20E-28	1.16E-27
IGFL2-AS1	0.021451	0.82744	5.269534	2.01E-07	2.43E-07
Z99572.1	1.842449	0.321199	−2.52009	1.21E-37	1.92E-36
AL135999.1	0.211246	0.893069	2.079847	7.64E-17	1.49E-16
AP000757.1	8.871118	1.183178	−2.90645	1.56E-36	1.47E-35
STAG3L5P-PVRIG2P-PILRB	0.259378	0.989455	1.931579	1.11E-18	2.88E-18
AC104564.3	0.177313	0.780917	2.138872	7.16E-18	1.56E-17
AC010618.3	0.27074	0.61849	1.191841	4.93E-13	7.11E-13
AC004923.4	0.157851	0.649031	2.039724	3.03E-22	1.16E-21
AC018521.5	1.243989	0.579176	−1.1029	2.72E-32	1.89E-31
LENG8-AS1	1.052021	3.302749	1.650504	3.37E-22	1.25E-21
AC090589.3	0.409017	1.357323	1.73053	4.99E-20	1.42E-19
AL132989.1	0.684699	1.931372	1.496084	3.45E-13	5.18E-13
AC105020.5	0.289828	1.055408	1.864533	2.15E-21	7.02E-21
HCG27	0.202245	1.578451	2.964331	3.72E-32	2.43E-31
AC092119.2	0.153225	0.659499	2.105717	8.33E-15	1.45E-14
AC148477.4	5.974009	0.803678	−2.89401	2.37E-34	1.88E-33
AC010201.2	0.34136	1.024145	1.585056	1.02E-12	1.43E-12
AP006621.2	0.441335	1.854161	2.07082	3.74E-14	6.10E-14
LINC00174	0.635957	1.856051	1.545236	1.25E-17	2.66E-17
LINC01612	3.150561	0.338203	−3.21965	2.61E-46	2.89E-44
AP003392.1	0.517678	1.75965	1.765161	2.38E-14	3.95E-14
AL353152.1	2.991248	0.170474	−4.13313	2.50E-39	5.77E-38
AC007566.1	0.641806	2.095451	1.707051	6.93E-18	1.54E-17
AL035661.1	29.66428	2.555783	−3.53689	1.54E-36	1.47E-35
AC012615.6	0.180508	0.822616	2.188153	7.62E-23	3.02E-22
COLCA1	3.252071	1.055255	−1.62377	7.27E-28	3.67E-27
AC109460.2	0.216085	0.790539	1.87124	7.14E-16	1.32E-15
AC135050.3	0.160602	1.482247	3.206223	1.68E-30	9.31E-30
AC006435.2	0.201304	0.937788	2.219884	2.41E-15	4.39E-15
AC005519.1	0.195832	0.735491	1.90909	9.31E-14	1.48E-13
LINC01094	0.27725	1.963631	2.824262	6.62E-37	9.18E-36
AC130469.1	0.057492	0.584919	3.346804	2.14E-19	5.80E-19
ATP6V0E2-AS1	1.195025	0.498375	−1.26174	1.62E-26	7.82E-26
AC002550.2	0.41261	0.988443	1.260379	1.49E-18	3.76E-18
AC002553.1	0.484197	1.375798	1.506603	2.07E-18	5.00E-18
AC007292.1	0.441184	0.99715	1.176431	2.88E-13	4.39E-13
AC067852.3	0.16725	0.609204	1.864922	6.88E-18	1.54E-17
AC015849.3	0.466717	1.710936	1.874166	3.75E-20	1.12E-19
AC022400.6	0.224677	0.581952	1.37305	2.67E-16	5.02E-16
SCAT2	0.178514	0.607967	1.767957	4.71E-08	5.75E-08
LINC02585	0.269199	0.702718	1.384273	8.69E-11	1.16E-10
SPINT1-AS1	9.524573	3.958528	−1.26669	1.02E-30	5.98E-30
AL022328.2	0.6408	2.063639	1.687244	2.97E-17	6.23E-17
MIR200CHG	2.760587	0.476474	−2.53451	1.27E-41	7.06E-40
SNHG20	1.119142	2.61343	1.223552	3.19E-25	1.36E-24

Abbreviations: logFC, log fold change; FDR: false discovery rate.

### lncRNA pairs and risk coefficient score models related to lipid metabolism

By virtue of an iterative loop and 0-or-1 matrixes, 1,510 aberrantly regulated lipid metabolism-associated lncRNA pairs were determined (Supplementary Table S2). As revealed in univariable cox regressive analysis, 25 lncRNA pairs could remarkably influence the OS of ccRCC patients. The aforesaid pairs were selected *via* a LASSO modeling method (Figures 2C, D). Consequently, 12 aberrantly regulated lipid metabolism-associated lncRNA pairs such as AC067817.2|AD001527.1, AD001527.1|COLCA1, AC093001.1|MCF2L-AS1, AL022328.1|AC002553.1, PRDM16-DT|IGFL2-AS1, AC009090.1|AC092119.2, AL031846.2|AC012615.6, AC015845.2|AC022400.6, AL162274.2|AC090589.3, AL031710.1|Z99572.1, AL662844.3|AC092119.2, and AC012615.6|SCAT2 were put into this risk model. Cox univariable regressive and Cox multivariable regressive analyses were completed on these 12 pairs of lipid metabolism-related lncRNAs (Figures 2E, F), and the risk coefficients of each pair of lipid metabolism-related lncRNA were acquired (Table 2).

**TABLE 2:** Analyses of regressive coefficients of 12 pairs of lipid metabolism-related lncRNAs to Cox associated with results.

lncRNA pairs	Coefficient	HR	HR.95L	HR.95H	*p*-value
AC067817.2|AD001527.1	−0.40245	0.668677	0.479532	0.932428	0.017674
AD001527.1|COLCA1	0.454706	1.575711	1.124176	2.208608	0.008305
AC093001.1|MCF2L-AS1	0.602433	1.826557	1.335812	2.497592	0.000161
AL022328.1|AC002553.1	−0.4151	0.660275	0.464762	0.938035	0.020503
PRDM16-DT|IGFL2-AS1	−0.42025	0.656885	0.48153	0.896096	0.007993
AC009090.1|AC092119.2	−0.45111	0.636923	0.457398	0.886909	0.007576
AL031846.2|AC012615.6	−0.48138	0.617933	0.393264	0.970952	0.036814
AC015845.2|AC022400.6	−0.4714	0.624128	0.42308	0.920714	0.017483
AL162274.2|AC090589.3	−0.35901	0.69837	0.507372	0.961267	0.027645
AL031710.1|Z99572.1	−0.42583	0.65323	0.475359	0.897657	0.008647
AL662844.3|AC092119.2	−0.27368	0.760574	0.540994	1.069277	0.115351
AC012615.6|SCAT2	−0.44327	0.641931	0.446515	0.922871	0.016696

Abbreviations: HR, hazard ratio; HR.95L: 95% CI, lower limit; HR.95H: 95% CI, upper limit.

### Assessment of the prognosis prediction ability of risk models

**FIGURE 3:**
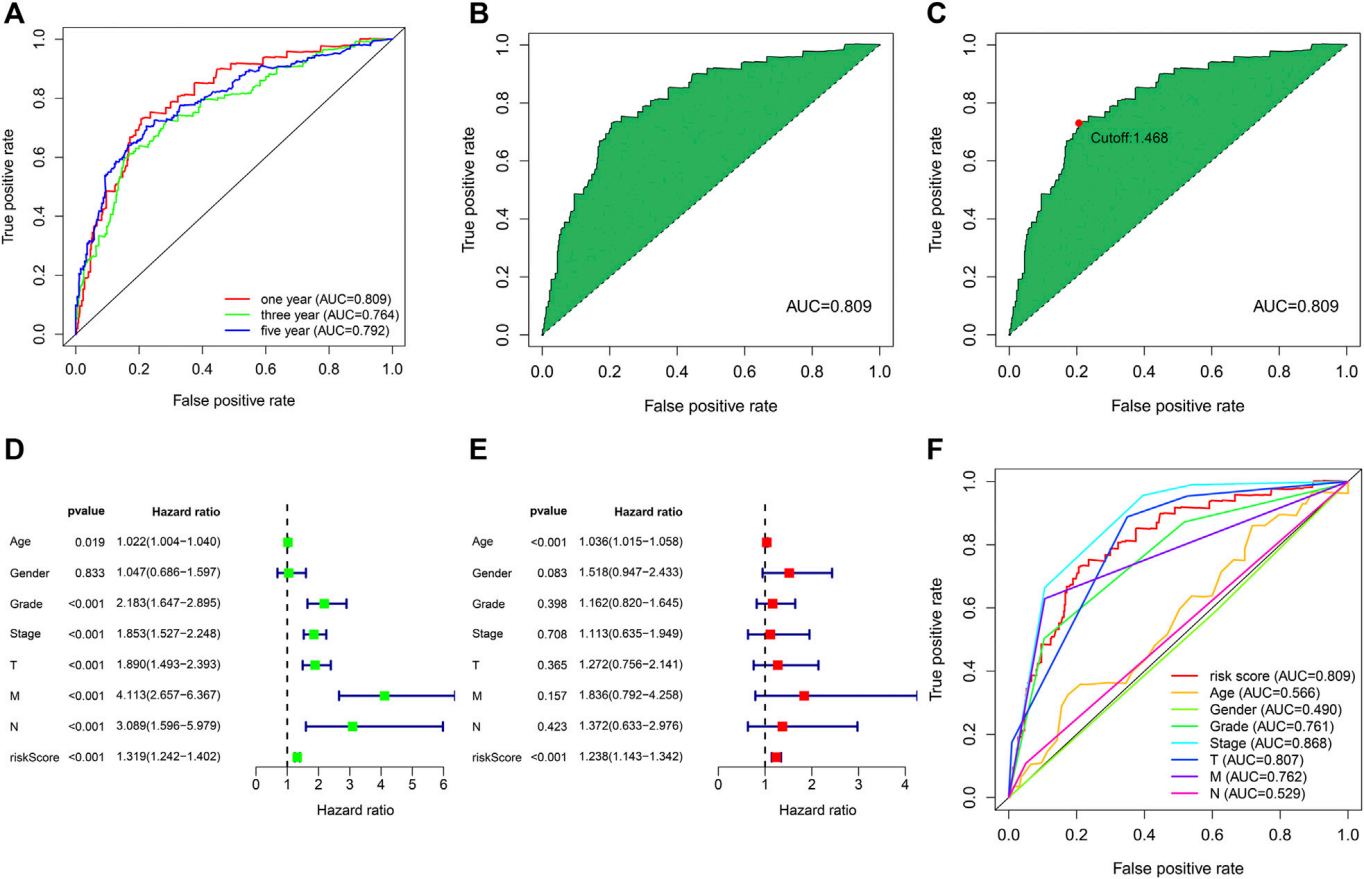
The assession of the prognosis prediction ability of risk models. **(A)** The 1-, 3- and 5-year ROC curves. **(B)** One-year ROC curve with maximal AUC result acquired based on the modeling method. **(C)** The cutoff value of 1.468 distinguishing risk_high_ and risk_low_ sufferers was acquired through the best fit. **(D)** Univariable cox regressive analysis of the associations of clinical-related indicators and risk score with the prognoses of ccRCC. **(E)** Multivariable cox regressive analysis of the associations of clinical-related indicators and risk score with the prognoses of ccRCC. **(F)** Comparison of the AUC values of clinical indicators and risk scores.

We used the above 12 pairs of prognostic lipid metabolism lncRNAs to construct the patient’s 1-, 3-, and 5-year ROC curves (Figure 3A), and discovered that the 1-year AUC was the largest (0.809) (Figure 3B). The 3- and 5-year AUC were 0.764 and 0.792, separately, which exhibited prediction ability as well. According to the optimum fit, the cutoff value to distinguish risk_high_ and risk_low_ sufferers was 1.468 (Figure 3C). We performed Cox univariable and multivariable regressive analyses on risk score and clinical indexes. Subsequently, by using the survival package of the R program, we visualized the data and drew the forest map (Figures 3D, E). In Cox univariate analysis, our team discovered that age, tumor grade, tumor stage, TNM stage and risk coefficient score were correlated with prognosis, while in Cox multivariable analyses, age and risk coefficient score were independent prognostic factors. The ROC curves for clinical indicators were compared with the 1-year risk coefficient score in the identical chart (Figure 3F). The results showed that risk coefficient score (AUC = 0.809) and tumor stage (AUC = 0.868) exhibited the greatest prediction ability.

### Risk models were used to analyze the correlation of clinical indicators

**FIGURE 4:**
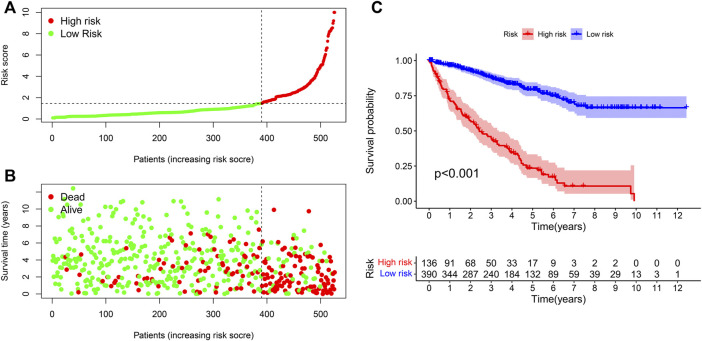
Correlation analysis of risk models and clinical indicators. **(A)** Computation of the risk score of every sufferer, so as to distinguish risk_high_ and risk_low_ sufferers as per the threshold. **(B)** Scatter plot of risk score and result for every sufferer. Red: high risk; Green: low risk. **(C)** K-M curve of OS between risk_high_ and risk_low_ sufferers.

We used the R program to study the association between risk coefficient score and ccRCC in the risk sub-group of sufferers (Figure 4A). As per the time process, the association between the survival status of ccRCC patients and risk coefficient score was acquired (Figure 4B), and a K-M curve was established on the foundation of the OS of risk_high_ and risk_low_ sufferers (Figure 4C). The survival rate in risk_low_ sufferers was remarkably better in contrast to risk_high_ sufferers (*p* < 0.001).

**FIGURE 5:**
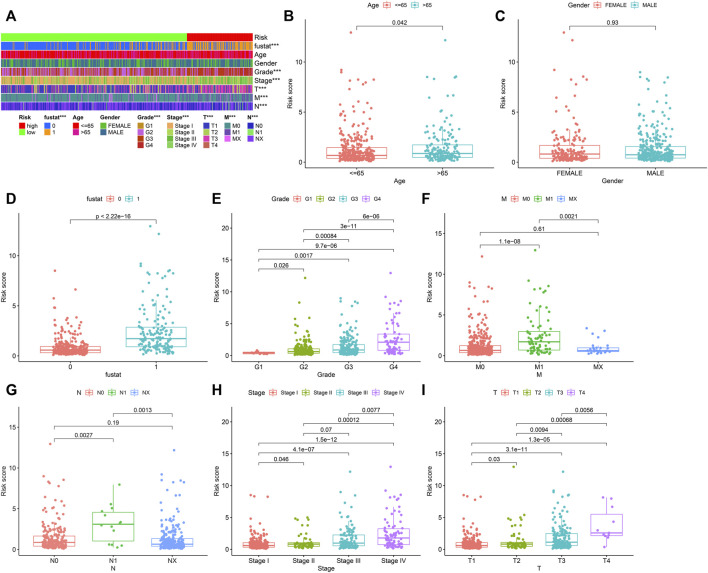
Collection of prognostic lipid metabolism-associated lncRNA hallmark and clinicopathologic features of ccRCC. **(A)** Heat maps of clinicopathologic features: survival status, age, gender, grade, stage, T, N and M in risk_high_ and risk_low_ sufferers. *p < 0.05; **p < 0.01; ***p < 0.001. Comparison of risk score in diverse clinicopathologic features: **(B)** age≤ 65 and> 65. **(C)** gender. **(D)** survival status (0: dead; 1: alive). **(E)** grade. **(F)** M. **(G)** N. **(H)** stage. **(I)** T.

The heat map in Figure 5A depicts the relation between risk score levels and clinical indexes. The result showed that age (*p* < 0.05), OS of ccRCC sufferers (*p* < 0.05), tumor grade (*p* < 0.05), M stage (*p* < 0.05), tumor stage (*p* < 0.05), T stage (*p* < 0.05), and N stage (*p* < 0.05) were significantly correlated with risk coefficient score. We can see from the box graph that the mortality of risk_high_ sufferers is greater (Figure 5D). In addition, age (Figure 5B), tumor grade (Figure 5E), M stage (Figure 5F), N stage (Figure 5G), tumor clinical stage (Figure 5H), and T stage (Figure 5I) were higher as well, while gender was not remarkably associated with risk coefficient score (Figure 5C).

### Association analyses of risk coefficient model and immune cell infiltration

**FIGURE 6:**
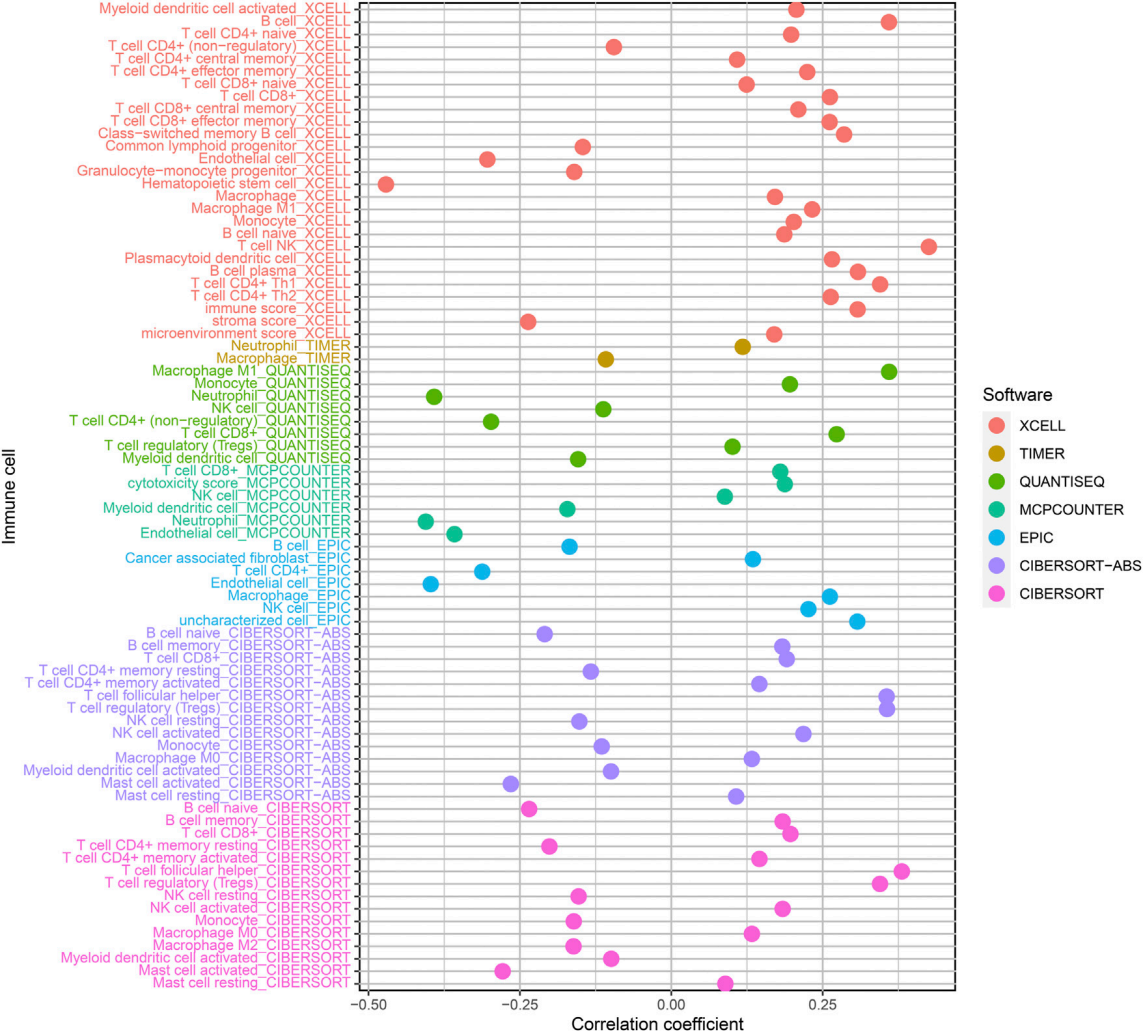
Relationship of risk score and immune cell infiltrations of ccRCC samples *via*: XCELL; TIMER; QUANTISEQ; MCPCOUNTER; EPIC; CIBERSORT-ABS and CIBERSORT.

Our team assessed the percentage of immune cells in specimens from these ccRCC patients *via* XCELL, TIMER, QUANTISEQ, MCPCOUNTER, EPIC, CIBERSORT-ABS, and CIBERSORT, based on marker genes and deconvolution algorithms. Meanwhile, the association between the risk coefficient model and infiltrating immunocytes was analyzed through the Pearson correlation test, and the screening criterion was *p* < 0.05. Data visualization was completed *via* the R program (Figure 6). The results indicated that the specimens from risk_high_ sufferers were positively related to NK cells, regulatory T cells and M1 macrophages infiltration in ccRCC, and related to neutrophil infiltration in a negative manner.

### Association analyses of risk coefficient model and genes

**FIGURE 7:**
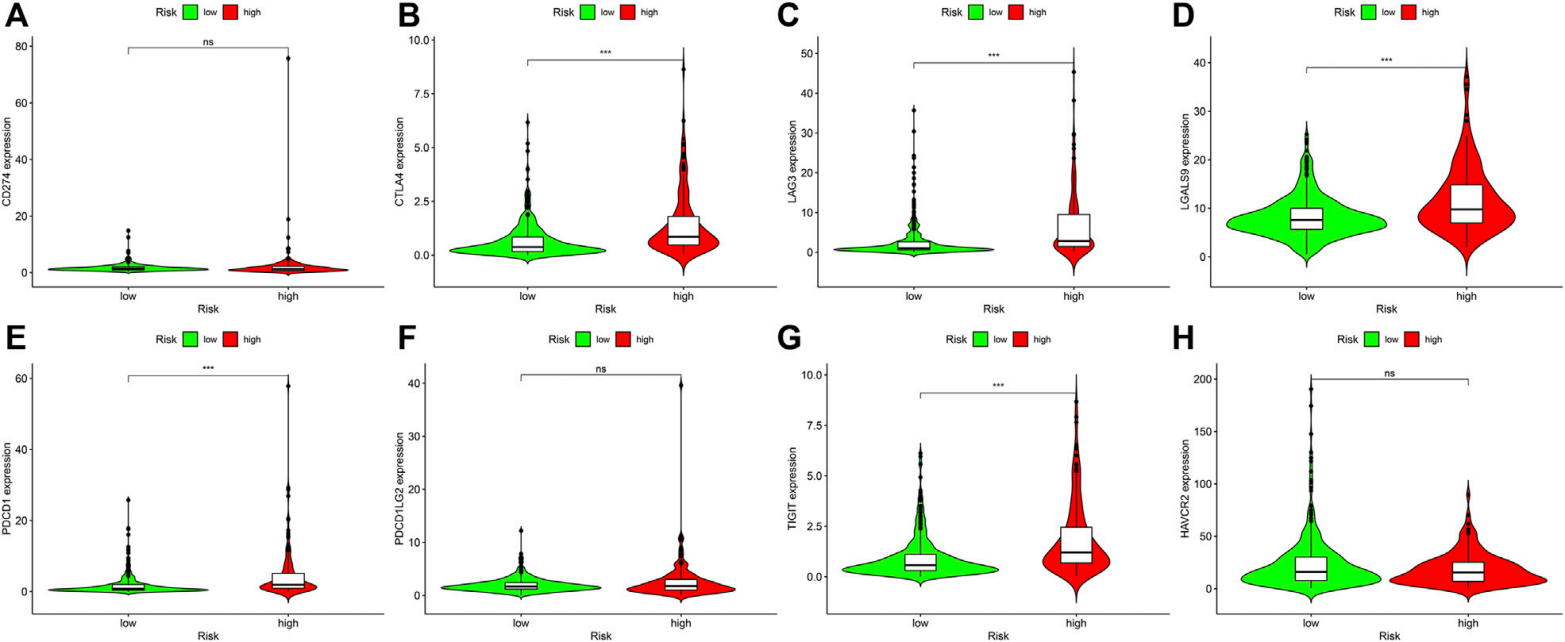
Associations between risk model and genes in ccRCC. Comparison of the expressing levels of **(A)** CD274. **(B)** CTLA4. **(C)** LAG3. **(D)** LGALS9. **(E)** PDCD1. **(F)** PDCD1LG2. **(G)** TIGIT. **(H)** HAVCR2. Ns: not signifcant; *p < 0.05; **p < 0.01; ***p < 0.001.

Targeted immune treatment is one of the most commonly used methods for treating ccRCC. In addition, our team investigated the relation between risk coefficient models and genes, and the result showed that amongst risk_high_ sufferers, the expressing levels of CTLA4 (*p* < 0.001; Figure 7B), LAG3 (*p* < 0.001; Figure 7C), LGALS9 (*p* < 0.001; Figure 7D), PDCD1 (*p* < 0.001; Figure 7E), and TIGIT (*p* < 0.001; Figure 7G) were elevated. The expressing levels of CD274 (*p* > 0.05; Figure 7A), PDCD1LG2 (*p* > 0.05; Figure 7F), and HAVCR2 (*p* > 0.05; Figure 7H) were slightly elevated. These genes may become potential novel diagnosis and treatment targets for ccRCC.

### Association analysis of risk coefficient model and targeted treatment medicines

**FIGURE 8:**
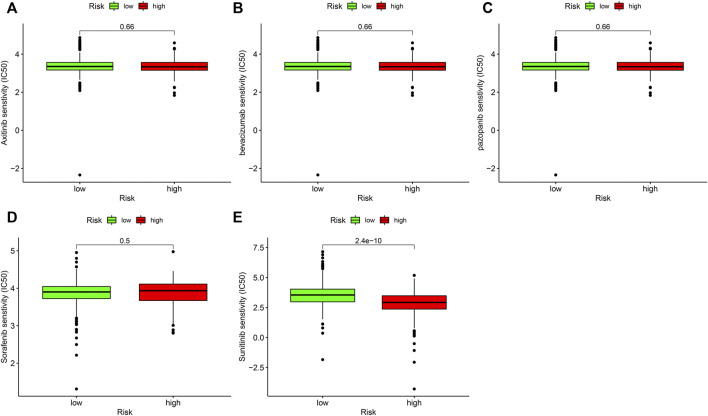
Associations between risk model and the susceptibility to chemo medicines. Comparison of the IC50 results of **(A)** axitinib. **(B)** bevacizumab. **(C)** pazopanib. **(D)** sorafenib and **(E)** sunitinib in risk_high_ and risk_low_ sufferers.

Target treatment is the first-line therapy for advanced ccRCC sufferers. The collection of risk coefficient score model and drug susceptibility of target treatment was analyzed. The curative effect of drugs was assessed through IC50. The lower the IC50, the higher the sensitivity of drugs. The result showed that risk_high_ sufferers were related to greater susceptibility to sunitinib (Figure 8E), which displayed statistical significance (*p* = 2.4e-10), while the sensitivity to axitinib (*p* = 0.66; Figure 8A), bevacizumab (*p* = 0.66; Figure 8B), pazopanib (*p* = 0.66; Figure 8C), and sorafenib (*p* = 0.5; Figure 8D) was not significantly different in risk_high_ and risk_low_ sufferers.

## Discussion

lncRNAs are non-coding RNAs that lack meaningful open reading frames and do not translate proteins, and their length is usually more than 200 nucleotide (Winkle et al., 2021). A large number of lncRNAs are transcribed by RNA polymerase Ⅱ and usually contain a poly adenosine tail structure with a complex spatial architecture (Liu et al., 2021). lncRNAs are widely distributed in the nucleus and cytoplasm, mostly expressed in the nucleus, and their surface is characterized by histological heterotropy (Li et al., 2021). Studies have shown that lncRNAs extensively participate in cell biological processes and regulate gene expression at multiple levels, including pre-transcription and post-transcription levels (Munteanu et al., 2021). lncRNAs have various biological functions, and their abnormal expressions are tightly associated with embryonic development and diseases, especially tumors (Ghafouri-Fard et al., 2021).

Recently, substantial researches have suggested that metabolic factors, such as obesity, dyslipidemia, and abnormal local lipid metabolism of tumors, are related to the onset and developmental process of RCC, especially the correlation between metabolic factors and the classification of renal clear cell carcinoma (Zhao et al., 2016; Lucarelli et al., 2020; Bobulescu et al., 2021). In previously finished researches, lncRNA-associated models of ccRCC were established as per the expressing levels of transcriptomic data. Herein, lipid metabolism-associated lncRNA pairs were used to establish a risk coefficient model to evaluate the prognosis of ccRCC sufferers, rather than according to the expressing levels of lncRNAs. TCGA was first utilized to obtain lncRNA and gene data related to lipid metabolism in ccRCC patients, and afterwards the R program was used to determine lncRNA associated with lipid metabolism. Subsequently, the differential expression of ccRCC and healthy neighboring specimens was analyzed to obtain the pairs of lncRNAs associated with lipid metabolism. Cox univariable analyses, multivariable regressive analyses and LASSO regressive analyses were used to obtain the risk coefficients of ccRCC patients in every specimen and establish the risk coefficient model. Based on the ROC curve, our team discovered that the 1-year AUC was the largest, and Akaike Information Criterion (AIC) was utilized to optimize fitting to acquire the cutoff value so as to distinguish risk_high_ and risk_low_ sufferers. The results of survival analyses of risk_high_ and risk_low_ sufferers revealed that the OS of risk_low_ sufferers was remarkably better in contrast to risk_high_ sufferers (*p* < 0.001).

In addition, our team assessed the relationship between risk coefficient score and clinical indicators for every specimen. Cox multivariate regressive analyses showed that age and risk coefficient score were independent prediction factors of prognosis. At the same time, ROC curves of clinical indexes were drawn to compare the ROC curves of 1-year risk factor scores in the identical chart. Our team discovered that 1-year risk factor score and were the optimum prediction factors of ccRCC, which revealed the dependability of our modeling method. There are also several models related to lipid metabolism. For example, Lu et al. (2022). have built a prognosis signature of lipid metabolism-related lncRNA in cervical cancer. The results showed that such lipid metabolism-related lncRNA model could facilitate the generation of new potential therapeutic targets for cervical cancer patients in clinical treatment and improve personalized treatment strategies, but the model was based on a single lncRNA rather than lncRNA pairs. Wang et al. (2022). built a model of metabolism-related lncRNA in hepatocellular carcinoma. They determined the prognosis features of nine characteristic lipid metabolism-related lncRNAs through research and verified the accurate type and reliability of the model through ROC, DCA and nomograph. The results showed that the prognostic model could help clinicians to provide personalized treatment strategies, however, their study did not analyze immune cell infiltration, immune checkpoint and drug prediction.

Our team utilized XCELL, TIMER, QUANTISEQ, MCPCOUNTER, EPIC, CIBERSORT-ABS, and CIBERSORT along with other methods to study the association between risky factor score and the data of immune cell infiltration, and correlation analysis was conducted as well. We discovered higher infiltration levels of NK cells, regulatory T cells, and M1 macrophages in risk_high_ sufferers.

It has been found that pD-L1+NK cells infiltrating tumor tissue are highly expressed in ccRCC sufferers. In addition, the results of *in vitro* cell experiments showed that NK cells could significantly inhibit the proliferative ability of CD8+T cells. These results suggested that NK tumor infiltration cells can weaken immunoregulation function (Sierra et al., 2021).

In addition, our team studied the association between immuno-checkpoint genes and the risk model of target treatment medicines, and discovered that the expressing levels of CTLA4, LAG3, LGALS9, PDCD1 and TIGIT were elevated in risk_high_ sufferers, while the expressing levels of CD274, PDCD1LG2 and HAVCR2 were slightly increased. These genes may become potential novel diagnosis and treatment targets for ccRCC.

ccRCC is the most commonly seen kidney carcinoma, taking up approximately 80 percent of all kidney cancers. About 30% of clear cell carcinoma of kidney is already advanced at the time of diagnosis, and about 10%–20% of early clear cell carcinoma will relapse and migrate after treatment (Bi et al., 2021). Currently, the therapy of advanced ccRCC is not satisfactory, and the 5-year OS is merely about 11.7% (Bedke et al., 2021). With the continuous development of various new drugs, the drug therapy of advanced renal clear cell carcinoma has evolved from the era of cell factors to the era of target medicines, and the current era of new immunodrug therapy (Yin et al., 2021). Targeted therapeutics, including vascular endothelial growth factor (VEGF) suppressors and mammalian target of rapamycin (mTOR), have been accepted due to the roles in treating advanced ccRCC, and have become the standard treatment (Zhang et al., 2021a). Recently, with the persistent development of immuno-checkpoint inhibitors (ICIs), the roles of ICIs in advanced ccRCC have been further confirmed, and it has become a kind of drug therapy for advanced renal clear cell carcinoma (Quhal et al., 2021). However, variations in the TME of ccRCC might be related to the occurrence of ccRCC tolerance to immunity-targeted medicines. Hence, sensitive medicines are accepted as they can reduce the cost of treatment and decrease the adverse effects of immunity-associated medicines. In the present paper, our team discovered that risk_high_ sufferers evidently displayed more sensitivity to sunitinib in contrast to risk_low_ sufferers, while the sensitivity to axitinib, bevacizumab, pazopanib, and sorafenib wasn’t remarkably diverse in risk_high_ and risk_low_ sufferers.

Although we utilized strict approaches and arithmetics to establish the model, this study still has some limitations. Firstly, because the lncRNA expression profiles of ccRCC patients with complete survival time are not available in public databases, this study did not have an external validation to evaluate the performance of the risk model. Therefore, it is necessary to acquire more clinical information and larger sample size to prove the dependability of the proposed risk coefficient model. At the same time, according to the results of this study, we believe that the risk coefficient model constructed by lipid metabolism-related lncRNAs can be further extended to other tumors and guide individualized treatment clinically.

## Conclusion

In summary, we identified new prognostic markers and established risk models for ccRCC, which could assess the prognosis of ccRCC patients and help determine which type of patients could be benefited from sunitinib. These discoveries will offer novel enlightenment regarding the diagnoses and therapies of ccRCC patients.

## Data Availability

The datasets presented in this study can be found in online repositories. The names of the repository/repositories and accession number(s) can be found in the article/Supplementary Material.

## Abbreviations

AIC, akaike information criterion; ccRCC, clear cell renal cell carcinoma; GTF, rene Transfer Format; ICIs, immuno-checkpoint inhibitors; K-M, Kaplan-Meier; lncRNAs, long non-coding RNAs; ncRNA, non-coding RNAs; RCC, renal cell carcinoma; VEGF, vascular endothelial growth factor.
